# A Retrospective observational cohort study of the effect of antenatal influenza vaccination on birth outcomes in Cape Town, South Africa, 2015‐2016

**DOI:** 10.1111/irv.12836

**Published:** 2021-01-16

**Authors:** Meredith L. McMorrow, Liza Rossi, Susan Meiring, Katherine Bishop, Raphaela Itzikowitz, Washiefa Isaacs, Faakhiera Stellenboom, Sibongile Walaza, Orienka Hellferscee, Florette K. Treurnicht, Heather J. Zar, Stefano Tempia, Cheryl Cohen

**Affiliations:** ^1^ Influenza Division Centers for Disease Control and Prevention Atlanta Georgia USA; ^2^ Influenza Program Centers for Disease Control and Prevention Pretoria South Africa; ^3^ United States Public Health Service Rockville Maryland USA; ^4^ Centre for Respiratory Diseases and Meningitis National Institute for Communicable Diseases of the National Health Laboratory Service Johannesburg South Africa; ^5^ Division of Public Health Surveillance and Response National Institute for Communicable Diseases National Health Laboratory Service Johannesburg South Africa; ^6^ Department of Paediatrics and Child Health Red Cross War Memorial Children’s Hospital Cape Town South Africa; ^7^ School of Public Health Faculty of Health Sciences University of the Witwatersrand Johannesburg South Africa; ^8^ School of Pathology Faculty of Health Sciences University of the Witwatersrand Johannesburg South Africa; ^9^ Medical Research Council Unit on Child & Adolescent Health University of Cape Town Cape Town South Africa; ^10^ MassGenics Duluth Georgia USA

**Keywords:** influenza vaccines, pregnancy outcome, premature birth

## Abstract

**Background:**

There are conflicting data concerning the impact of antenatal influenza vaccination on birth outcomes including low birthweight (LBW), preterm birth, small for gestational age (SGA), and stillbirth.

**Methods:**

We conducted a retrospective observational cohort study of infants born to women residing in Mitchells Plain, Cape Town. Infants were born at 4 health facilities during May 28 – December 31, 2015 and April 15 – December 31, 2016. We performed crude and multivariable logistic regression, propensity score (PS) matching logistic regression, and inverse probability of treatment weighted (IPTW) regression to assess vaccine effectiveness (VE) against LBW, preterm birth, SGA, and stillbirth adjusting for measured confounders.

**Results:**

Maternal vaccination status, antenatal history, and ≥1 birth outcome(s) were available for 4084/5333 (76.6%) pregnancies, 2109 (51.6%) vaccinated, and 1975 (48.4%) unvaccinated. The proportion LBW was lower in vaccinated (6.9%) vs. unvaccinated (12.5%) in multivariable [VE 0.27 (95% CI 0.07‐0.42)], PS [VE 0.30 (95% CI 0.09‐0.51)], and IPTW [VE 0.24 (95% CI 0.04‐0.45)]. Preterm birth was less frequent in vaccinated (8.6%) than unvaccinated (16.4%) in multivariable [VE 0.26 (0.09‐0.40)], PS [VE 0.25 (95% CI 0.09‐0.41)], and IPTW [VE 0.34 (95% CI 0.18‐0.51)]. The proportion SGA was lower in vaccinated (6.0%) than unvaccinated (8.8%) but not in adjusted models. There were few stillbirths in our study population, 30/4084 (0.7%).

**Conclusions:**

Using multiple analytic approaches, we found that influenza vaccination was associated with lower prevalence of LBW (24‐30%) and preterm birth (25‐34%) in Cape Town during 2015‐2016.

## 1 Introduction

Seasonal and pandemic influenza virus infections are associated with severe disease outcomes in pregnant women and young infants.^1^, ^2^, ^3^, ^4^, ^5^, ^6^ Maternal antenatal influenza vaccination reduces the incidence of influenza among pregnant women and their young infants during the first 4‐6 months of life.^7^, ^8^, ^9^, ^10^, ^11^ In 2012, the World Health Organization Strategic Advisory Group of Experts on Immunization recommended prioritization of pregnant women for influenza vaccination.^12^ Since that time, several countries have introduced influenza vaccination programs including programs that target pregnant women^13^; however, this recommendation has not been widely implemented in sub‐Saharan Africa.^14^

There have also been conflicting data concerning the potential impact of antenatal influenza vaccination on birth outcomes including stillbirth, preterm birth, and low birth weight. Influenza pandemics have been associated with fetal loss,^15^, ^16^, ^17^ preterm birth,^16^, ^18^ and infants born small for gestational age^18^ particularly following maternal influenza‐associated or acute respiratory hospitalization; however, there appears to be limited association between seasonal influenza epidemics and birth outcomes.^19^, ^20^, ^21^, ^22^ A small randomized, controlled trial (RCT) of maternal influenza immunization in Bangladesh^23^ found that maternal influenza immunization increased mean birth weight following the influenza season and a larger RCT in Nepal^24^ found that year‐round maternal influenza immunization reduced the proportion of infants born low birth weight, but similar trials in South Africa^9^ and Mali^10^ found no impact on these measures. Likewise, observational studies of the impact of maternal influenza vaccination on birth outcomes including stillbirth, preterm birth, low birth weight, or small for gestational age have not provided conclusive evidence of association^25^, ^26^, ^27^, ^28^ and may be subject to bias introduced in vaccination or reporting of comorbidities/risk factors when an adverse birth outcome occurs.

Since 2010, South Africa has offered seasonal influenza vaccination at no cost at public health facilities.^29^ Approximately 1 million doses of Southern Hemisphere trivalent inactivated influenza vaccine are procured annually to target risk groups including pregnant women, children aged 6‐59 months, healthcare workers, persons aged 65 years or older and persons with chronic illness including HIV and tuberculosis. An additional 1 million doses are available through the private sector. South Africa is a middle‐income country with a high antenatal prevalence of HIV (30.7% (95% CI: 30.1%−31.3%) nationally; 15.9% (95% CI: 14.2%–17.8%) in the Western Cape Province (WCP) in 2017^30^), and an annual birth cohort of approximately 1.2 million.^31^ In 2016, South Africa’s National Advisory Group on Immunization (NAGI) further recommended that pregnant women and persons with HIV infection should be prioritized for seasonal influenza vaccination.^32^ Despite this prioritization, influenza vaccine coverage remains <16% in pregnant women.^32^ As part of a study of maternal influenza vaccine effectiveness against infant influenza‐associated hospitalization, we collected data on adverse birth outcomes among infants born to women eligible for antenatal influenza vaccination during 2015 and 2016 at 4 health facilities in Cape Town to assess the effect of maternal antenatal influenza vaccination on birth outcomes.

## 2 Methods

### 2.1 Study design and population

In South Africa, most antenatal care is provided at primary health centers and maternal obstetric units. Women with chronic illnesses, prior pregnancy complications including cesarean section, or who develop high‐risk conditions during pregnancy are referred to district hospitals or regional referral hospitals for continuation of antenatal care. We conducted a retrospective observational cohort study of infants born to women residing in Mitchells Plain, a suburb of Cape Town. These infants were born at 4 health facilities (Mitchells Plain Maternal Obstetric Unit, Mitchells Plain District Hospital, Mowbray Maternity Hospital and Groote Schuur Hospital) representing the continuum of care for pregnant women in Mitchells Plain during May 28 – December 31, 2015 and April 15 – December 31, 2016. We collected data on birth outcomes for infants born to women who sought antenatal care during influenza vaccination campaigns during May 14 – August 16, 2015 or April 1 – August 25, 2016. Influenza vaccines were offered at no cost at all public antenatal clinics except Groote Schuur Hospital’s antenatal clinic where most high‐risk pregnancies are referred for care. Maternal age, parity, HIV status, medical history, number of antenatal visits, tobacco, and alcohol use were abstracted from antenatal records or birth registers.

### 2.2 Primary outcome measures

We used influenza surveillance from local general practitioners in the WCP (from the Viral Watch Programme^33^) to monitor influenza activity during the study period. Birth outcomes of interest included stillbirth (fetal death after 20 weeks gestation), mean birthweight, low birthweight (LBW) (birthweight less than 2500 g), preterm birth (birth before 37 weeks gestation), and small for gestational age (SGA) (birthweight <10th percentile for gestational age using WHO fetal growth charts^34^). Gestational age was determined by the clinician from last menstrual period, antenatal ultrasound, or fundal height at first antenatal visit. Birthweight was recorded at the time of delivery in grams.

### 2.3 Vaccination campaign and determination of influenza vaccination status

Influenza vaccines are offered in limited supply at no cost in public antenatal clinics. Influenza vaccines are also offered by private general practitioners and at local pharmacies. During the period of this study, we increased influenza vaccine availability at selected clinics to increase coverage as part of a larger study of the effectiveness of maternal influenza vaccination against infant influenza‐associated hospitalization. As part of the study, we kept influenza vaccine registers at study clinics to document vaccine uptake and encouraged documentation of influenza vaccination status of pregnant women in antenatal records and birth registers. We reviewed antenatal records, birth registers, and vaccine registers at clinics to determine maternal vaccination status. Women who did not receive antenatal care and those who received care at clinics that did not provide influenza vaccination were considered unvaccinated in all analyses. Women who had not entered the second trimester prior to the end of the influenza vaccine campaign and who were not listed in clinic vaccine registers were also considered unvaccinated. Women who received influenza vaccination less than 2 weeks prior to delivery or whose date of vaccination was outside the campaign period were excluded from the primary analyses.

### 2.4 Determination of maternal HIV status

Healthcare workers offered HIV testing of pregnant or postpartum women according to the standard practice for the healthcare facility. Typically, pregnant women were screened by rapid HIV test with confirmation by HIV ELISA if rapid HIV test was positive. Pregnant women received HIV testing and counseling throughout pregnancy including repeat testing of all previously HIV‐uninfected women at the time of delivery per national guidelines.

### 2.5 Statistical analysis

We described the characteristics of vaccinated and unvaccinated women and their infants using frequencies for categorical variables and means for continuous variables. We used Pearson *Χ* ^2^ and Wald *Χ* ^2^ to assess differences in categorical variables, and two‐sample t test for differences in means. We performed multivariable logistic regression to assess vaccine effectiveness against low birth weight, preterm birth, small for gestational age and stillbirth adjusting for measured confounders. We calculated vaccine effectiveness (VE) as 1‐odds ratio (OR) for all logistic regression analyses.

We also explored other methods to adjust for observational bias using methodology similar to Walsh et al.^35^ We used standardized differences to assess the balance of baseline covariates between the vaccinated and unvaccinated groups and considered an absolute standardized difference below 0.10 indicative of a balanced covariate. We included the following variables in logistic regression propensity score models: maternal age, campaign year, season of birth, site, gravidity, parity, maternal HIV status, non‐HIV chronic illness, smoking, alcohol use, and frequency of antenatal care attendance. Non‐HIV chronic illness included anemia (n = 246), pregnancy induced hypertension (n = 184), hypertension (n = 120), asthma (n = 118), diabetes (n = 76), obesity (n = 67), psychiatric disorders (n = 20), heart disease (n = 18), seizures (n = 12), and other comorbidities (n = 98). We then developed inverse probability of treatment weights (IPTWs), which weighted vaccinated pregnancies by the inverse of the propensity score and unvaccinated pregnancies by the inverse of one minus the propensity score. To generate adjusted results, we ran propensity score matching logistic regression models and IPTW weighted logistic regression models to assess the average treatment effect in the treated (ATET) for each outcome except birthweight. Complete data were available on 3869/4084 (94.7%) of pregnancies for inclusion in propensity score analyses. We assessed the impact of vaccination on birth weight and adjusted for measured confounders using inverse probability weighted (IPW) linear regression.

Because we had limited data about outcomes of prior pregnancies, we created additional propensity score models for only primigravidae including all listed variables except parity. IPTWs were assigned using the same methodology but with the propensity score developed for primigravidae. Complete data were available for 1218/1286 (94.7%) of primigravidae for propensity score development.

### 2.6 Ethical review

The University of Witwatersrand Human Research Ethics Committee (certificate M140826) and the Faculty of Health Sciences, University of Cape Town Human Research Ethics Committee (ref 835_2014) approved the study protocol. The National Health Research Committee and the Western Cape Provincial Health Research Committee also approved the protocol. The US Centers for Disease Control and Prevention relied on the local ethical review (CDC protocol #6746).

## 3 Results

### 3.1 Influenza activity

**FIGURE 1:**
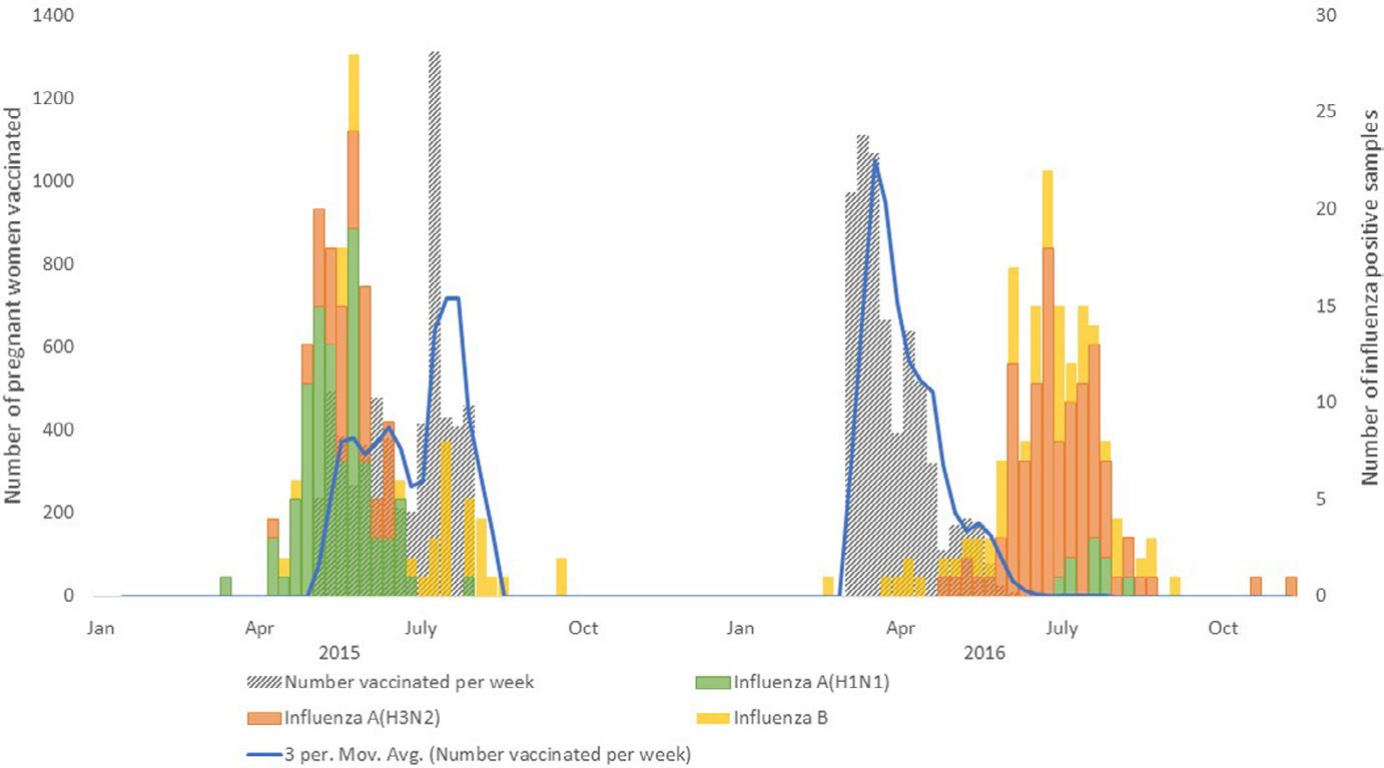
Influenza activity and number of women vaccinated by week, Cape Town, South Africa, 2015‐2016

In 2015, the influenza season began in WCP in week 17 (week ending May 2nd) and ended in week 36 (Figure 1). Of 326 WCP samples tested in the Viral Watch Programme, 173 (53%) tested positive for influenza viruses: 95 (55%) influenza A(H1N1)pdm09, 43 (25%) influenza A(H3N2), and 35 (20%) influenza B viruses. Influenza activity peaked in week 23. In 2016, the influenza season began in week 19 (week ending May 13th) and ended in week 40 (Figure 1). Of the 280 samples tested from the WCP in the Viral Watch Programme, 167 (60%) tested positive for influenza viruses: 105 (63%) for influenza A(H3N2), 53 (32%) for influenza B, and 9 (5%) for influenza A(H1N1)pdm09 viruses. Influenza activity peaked in week 31.

### 3.2 Vaccination campaigns

Due to manufacturing challenges, there was a delay in receiving Southern Hemisphere influenza vaccines in 2015.^36^ The maternal influenza vaccination campaign began on May 14th during week 20, less than three weeks before influenza activity peaked. During May 14th‐August 16th, 2015, 6058 pregnant women were vaccinated in clinics serving the catchment area of the study sites. Influenza A(H1N1)pdm09 was the predominant circulating strain, and there was no vaccine mismatch. In 2016, the vaccine campaign began on April 1st during week 14, and 6461 pregnant women were vaccinated before August 25th, 2016. All but 6 (0.1%) women were vaccinated more than two weeks before the peak of the season. Influenza A(H3N2) was the predominant circulating strain and vaccine effectiveness was 18% (95% CI −55% to 57%) due to poor match with the A(H3N2) strain.^33^

### 3.3 Vaccine coverage and baseline characteristics

**FIGURE 2:**
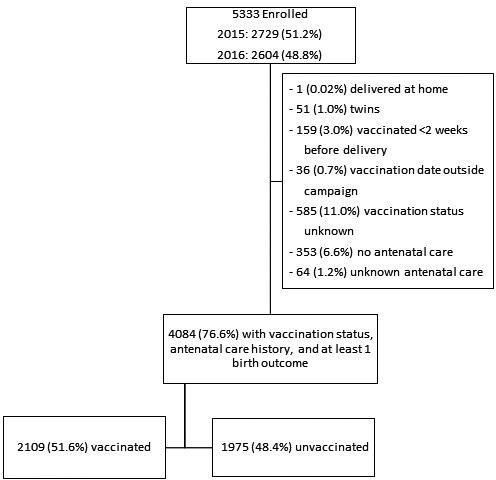
Summary of enrolled mothers who received antenatal influenza vaccination and those who did not receive antenatal influenza vaccination with data available on at least one birth outcome, Cape Town, South Africa, 2015‐2016

We collected data on birth outcomes for 5333 pregnancies during the study period (Figure 2). Maternal vaccination status, antenatal history, and one or more birth outcomes were available for 4084 (76.6%) pregnancies. Among these 4084 women, 2109 (51.6%) were vaccinated and 1975 (48.4%) were unvaccinated (Table 1). Among 2069 (98.1%) vaccinated women with information on gestational age at the time of vaccination, 139 (6.7%) were vaccinated in the first trimester, 992 (48.0%) in the second trimester, and 938 (45.3%) in the third trimester. HIV status was available for 4081 (99.9%) women and 428 (10.5%) were HIV‐infected. There were statistically significant differences (*P *< 0.05) between vaccinated and unvaccinated women in maternal age, season of infant birth, site, gravidity, parity, number of antenatal care visits, and non‐HIV chronic illness. On multivariable analysis, site, parity, season of infant birth, and number of antenatal care visits remained significantly associated with vaccination. The standardized difference in baseline characteristics was greater than 0.10 for season of infant birth, gravidity, parity, number of antenatal visits, and non‐HIV chronic illness. The IPTW weighted absolute standardized differences were smaller but did not completely correct for these differences, especially the differences in antenatal care visits and season of infant birth.

**TABLE 1:** Demographic characteristics of postpartum women by influenza vaccination status, N=4084, Cape Town, South Africa, 2015‐2016

Characteristic	All N = 4084	Vaccinated n = 2109 (51.6%)	Unvaccinated n = 1975 (48.4%)	*P* value	Standardized difference	IPTW weighted standardized difference^§^
Maternal age in years, n/N (%)						
<20	440 (10.8)	217 (10.3)	223 (11.3)	0.022	0.055	0.043
20‐24	1196 (29.3)	660 (31.3)	536 (27.1)			
25‐29	1177 (28.8)	605 (28.7)	572 (29.0)			
30‐34	794 (19.4)	403 (19.1)	391 (19.8)			
≥35	477 (11.7)	224 (10.6)	253 (12.8)			
Mean age in years (range)	26.7 (13‐47)	26.5 (13‐45)	27.0 (13‐47)	0.013		
Campaign year, n (%)						
2015	2195 (53.7)	1113 (52.8)	1082 (54.8)	0.198	0.059	0.079
2016	1889 (46.3)	996 (47.2)	893 (45.2)			
Season of infant birth, n (%)						
Before influenza season	327 (8.0)	172 (8.2)	155 (7.8)	<0.001	0.341	0.323
During influenza season	1130 (27.7)	801 (38.0)	329 (16.7)			
After influenza season	2627 (64.3)	1136 (53.9)	1491 (75.5)			
Site, n (%)						
Mitchell’s Plain Maternal	1436 (35.2)	745 (35.3)	691 (35.0)	<0.001	0.007	0.014
Obstetric Unit						
Mitchell’s Plain District Hospital	1633 (40.0)	937 (44.4)	696 (35.2)			
Mowbray Maternity Hospital	714 (17.5)	337 (16.0)	377 (19.1)			
Groote Schuur Hospital	301 (7.4)	90 (4.3)	211 (10.7)			
Pregnancy history						
Gravidity, n/N (%)						
1	1286/4082 (31.5)	701/2109 (33.2)	585/1973 (29.7)	<0.001	0.146	0.115
2‐3	2103/4082 (51.5)	1111/2109 (52.7)	992/1973 (50.3)			
4 or more	693/4082 (17.0)	297/2109 (14.1)	396/1973 (20.1)			
Parity^†^, n/N (%)						
0‐1	1542/4080 (37.8)	840/2107 (39.9)	702/1973 (35.6)	<0.001	0.158	0.117
2‐3	2079/4080 (51.0)	1086/2107 (51.5)	993/1973 (50.3)			
4 or more	459/4080 (11.3)	181/2107 (8.6)	278/1973 (14.1)			
Antenatal visits, n/N (%)						
1‐3	1104 (27.0)	354 (16.8)	750 (38.0)	<0.001	0.458	0.398
4‐6	2147 (52.6)	1231 (58.4)	916 (46.4)			
7 or more	833 (20.4)	524 (24.8)	309 (15.6)			
HIV‐infected, n (%)	428/4081 (10.5)	205/2108 (9.7)	223/1973 (11.3)	0.100	0.039	0.032
CD4 <200, n (%)	44/393 (11.2)	20/192 (10.4)	24/201 (11.9)	0.632		
Antiretroviral Therapy (ART) use among HIV‐infected, n (%)	410/415 (98.8)	202/202 (100)	208/213 (97.7)	0.028		
Non‐HIV chronic illness^‡^, n (%)	954/3939 (24.2)	441/2018 (21.9)	513/1921 (26.7)	<0.001	0.116	0.125
Smoked during pregnancy, n (%)	1369/4025 (34.0)	683/2083 (32.8)	686/1942 (35.3)	0.090	0.065	0.046
Alcohol during pregnancy, n (%)	376/4023 (9.3)	193/2082 (9.3)	183/1941 (9.4)	0.863	0.007	0.016
Trimester of vaccination, n (%)						
First		139/2069 (6.7)				
Second		992/2069 (48.0)				
Third		938/2069 (45.3)				

^†^ Enrolled patients based on delivery record therefore parity reflects outcome of pregnancy under study.

^‡^ Non‐HIV chronic illness included anemia (n = 246), pregnancy‐induced hypertension (n = 184), hypertension (n = 120), asthma (n = 118), diabetes (n = 76), obesity (n = 67), psychiatric disorders (n = 20), heart disease (n = 18), seizures (n = 12), and other comorbidities (n = 98).

^§^ Inverse probability of treatment weighted (IPTW) regression adjusted for maternal age, site, season of birth, campaign year, gravidity, parity, number of antenatal visits, maternal HIV status, chronic illness, smoking, and alcohol use during pregnancy.

### 3.4 Birth outcomes

The mean birth weight among live births included in our analyses (n = 4053) was 3150g (95% CI 3133 to 3167) and was statistically significantly higher in vaccinated compared to unvaccinated women using crude linear regression [114g difference (95% CI 80 to 148), *P *< 0.001] and IPW regression [36g difference (95% CI 1 to 70), *P* = 0.04], but not multivariable linear regression [20g difference (95% CI −11 to 51), *P* = 0.20](Table 2). Analyses limited to primigravidae also found a significant difference in mean birth weight between vaccinated and unvaccinated women using crude linear regression [104g difference (95% CI 46 to 162), *P* < 0.001], but no significant difference remained after adjustment in multivariable linear regression (*P* = 0.22) or IPW regression (*P* = 0.10)(Table 3).

**TABLE 2:** Analysis of birth outcomes by maternal vaccination status and influenza exposure, N = 4084, Cape Town, South Africa, 2015‐2016

Outcome	All N = 4084	Vaccinated n = 2109 (51.6%)	Unvaccinated n = 1975 (48.4%)	Crude Weight Difference or Odds Ratio in Vaccinated vs Unvaccinated (95% CI)	Adjusted Effect in Vaccinated vs Unvaccinated (95% CI)	Propensity score matched Effect in Vaccinated vs Unvaccinated (95% CI)	Inverse probability weighted Effect in Vaccinated vs Unvaccinated (95% CI)
Mean birth weight^†^, g (95% CI)	3150 (3133‐3167)	3205 (3183‐3227)	3091 (3064‐3117)	114 (80 to 148)	20 (−11 to 51)^‡^ ^a^	NA	36 (1 to 70)^§^
Proportion low birth weight^†^, n/N (%)	390/4053 (9.6)	146/2102 (6.9)	244/1951 (12.5)	0.52 (0.42 to 0.65)	0.27 (0.07 to 0.42)^b^	0.30 (0.09 to 0.51)	0.24 (0.04 to 0.45)
Proportion preterm^†^, n/N (%)	500/4051 (12.3)	180/2101 (8.6)	320/1950 (16.4)	0.48 (0.39 to 0.58)	0.26 (0.09 to 0.40)^b^	0.25 (0.09 to 0.41)	0.34 (0.18 to 0.51)
Proportion small for gestational age^†^, n/N (%)	298/4049 (7.4)	126/2101 (6.0)	172/1948 (8.8)	0.66 (0.52 to 0.84)	0.15 (−0.10 to 0.34)^b^	0.15 (−0.08 to 0.38)	0.22 (−0.01 to 0.45)
Proportion stillbirth, n/N (%)	30/4084 (0.7)	6/2109 (0.3)	24/1975 (1.2)	0.23 (0.09 to 0.57)	0.62 (0.04 to 0.85)^c^	0.61 (−0.07 to 1.30)	0.49 (−0.25 to 1.24)

^a^ Adjusted for gestational age, site, parity, number of antenatal visits, maternal HIV status, and smoking during pregnancy.

^b^ Adjusted for site, parity, number of antenatal visits, and smoking during pregnancy.

^c^ Adjusted for campaign year, site, and number of antenatal visits.

^†^ low birthweight (birthweight less than 2500 g), preterm birth (less than 37 weeks gestational age), and small for gestational age (birthweight <10^th^ percentile for gestational age using WHO fetal growth charts) among live births with birth weight reported (n = 4053).

^‡^ linear regression coefficient for birth weight which is a continuous variable (n = 3986).

^§^ inverse probability weighting with regression adjustment (n = 3837).

**TABLE 3:** Analysis of birth outcomes by maternal vaccination status and influenza exposure among primigravidae, N = 1286, Cape Town, South Africa, 2015‐2016

Outcome	All N = 1286	Vaccinated n = 701 (54.5%)	Unvaccinated n = 585 (45.5%)	Crude Weight Difference or Odds Ratio in Vaccinated vs Unvaccinated (95% CI)	Adjusted Effect in Vaccinated vs Unvaccinated (95% CI)	Propensity score matched Effect in Vaccinated vs Unvaccinated (95% CI)	Inverse probability weighted Effect in Vaccinated vs Unvaccinated (95% CI)
Mean birth weight^†^, g (95% CI)	3116 (3087‐3200)	3163 (3126‐3200)	3059 (3013‐3105)	104 (46 to 162)	32 (−20 to 84) ^†^	NA	48 (−9 to 105)^§^
Proportion low birth weight†, n/N (%)	119/1275(9.3)	49/700 (7.0)	70/575 (12.2)	0.54 (0.37 to 0.80)	0.27 (−0.11 to 0.52)^b^	0.03 (−0.01 to 0.07)	0.03 (−0.01 to 0.06)
Proportion preterm^†^, n/N (%)	161/1275 (12.6)	71/700 (10.1)	90/575 (15.7)	0.61 (0.44 to 0.85)	0.08 (−0.33 to 0.36)^c^	0.13 (−0.18 to 0.43)	0.16 (−0.14 to 0.45)
Proportion small for gestational age†, n/N (%)	98/1273 (7.7)	45/700 (6.4)	53/573 (9.2)	0.67 (0.45 to 1.02)	0.14 (−0.33 to 0.44)^b^	0.15 (−0.26 to 0.38)	0.15 (−0.24 to 0.55)
Proportion stillbirth, n/N (%)	11/1286 (0.9)	1/701 (0.1)	10/585 (1.7)	0.08 (0.01 to 0.64)	0.89 (0.15 to 0.99)^c^	0.77 (0.13 to 1.41)	0.83 (0.03 to 1.63)

^a^ Adjusted for gestational age, site, number of antenatal visits, and smoking during pregnancy.

^b^ Adjusted for site, number of antenatal visits, and smoking during pregnancy.

^c^ Adjusted for site and number of antenatal visits.

^†^ low birthweight (birthweight less than 2500 g), preterm birth (less than 37 weeks gestational age), and small for gestational age (birthweight <10^th^ percentile for gestational age using WHO fetal growth charts) among live births with birth weight reported (n = 1275).

^‡^ linear regression coefficient for birth weight which is a continuous variable (n = 1250).

^§^ inverse probability weighting with regression adjustment (n = 1207).

Among 4053 births, 390 (9.6%) were low birthweight (<2500g). The proportion low birthweight was significantly different between vaccinated (146/2102; 6.9%) and unvaccinated (244/1951; 12.5%) women on crude logistic regression [OR 0.52 (95% CI 0.42‐0.65), *P* < 0.001] and remained so after adjustment using multivariable logistic regression [VE 0.27 (95% CI 0.07‐0.42), *P* = 0.009], propensity score matching logistic regression [VE 0.30 (95% CI 0.09‐0.51), *P *= 0.005], and IPTW weighted logistic regression [VE 0.24 (95% CI 0.04‐0.45), *P* = 0.02] (Table 2). However, in analyses limited to primigravidae an initial difference identified in the crude odds ratio [OR 0.54 (95% CI 0.37‐0.80), *P* = 0.002] was not significantly different in any of the 3 adjusted models, *P* = 0.14, 0.10, and 0.116, respectively (Table 3).

Preterm birth was the most commonly observed adverse birth outcome, 500/4051 (12.3%) (Table 2). Preterm birth was significantly less frequent in vaccinated (180/2101; 8.6%) than unvaccinated women (320/1950; 16.4%) on crude logistic regression [OR 0.48 (95% CI 0.39‐0.58), *P* < 0.001] and remained so in all adjusted models: multivariable logistic regression [VE 0.26 (95% CI 0.09‐0.40), *P* = 0.005], propensity score matched logistic regression [VE 0.25 (95% CI 0.09‐0.41), *P* = 0.003], and IPTW weighted logistic regression [VE 0.34 (95% CI 0.18‐0.51), *P* < 0.001]. Preterm birth was also commonly reported among primigravidae, 161/1275 (12.6%) (Table 3). Preterm birth was less frequent in vaccinated primigravidae (71/700; 10.1%) than unvaccinated primigravidae (90/575; 15.7%) on logistic regression analysis [OR 0.61 (95% CI 0.44‐0.85), *P *= 0.003] but this association did not remain significant in any of the 3 adjusted models, *P* = 0.67, 0.42, and 0.31, respectively.

Overall, the proportion of live births small for gestational age was 7.4% (298/1273). The proportion of live births small for gestational age was statistically significantly lower in vaccinated (126/2101; 6.0%) than unvaccinated (172/1948; 8.8%) women [OR 0.66 (95% CI 0.52‐0.84), *P* = 0.001] on crude logistic regression but did not remain so in any of the 3 adjusted models, *P* = 0.22, 0.19, 0.06, respectively (Table 2). SGA was not significantly associated with vaccination status among primigravidae on logistic regression or adjusted analyses, *P* = 0.06, 0.50, 0.48, and 0.44, respectively (Table 3).

There were very few stillbirths documented in our study population, 30/4084 (0.7%) (Table 2). Reporting did not consistently denote fresh or macerated stillbirth. While stillbirth was less common in vaccinated (6/2109; 0.3%) compared to unvaccinated (24/1975; 1.2%) women on crude logistic regression [OR 0.23 (95% CI 0.09‐0.57), *P* = 0.001] and multivariable logistic regression [VE 0.62 (95% CI 0.04‐0.85), *P* = 0.04], there was no significant association in propensity score matched (*P* = 0.08) or IPTW weighted logistic regression (*P* = 0.19) (Table 2). Eleven (0.9%) stillbirths were reported among 1286 primigravidae (Table 3). Stillbirth was less common in vaccinated (1/701; 0.1%) compared to unvaccinated (10/585; 1.7%) primigravidae on crude logistic regression [OR 0.08 (95% CI 0.01‐0.64), *P* = 0.02], multivariable logistic regression [VE 0.89 (95% CI 0.15‐0.99), *P* = 0.04], propensity matched logistic regression [VE 0.77 (95% CI 0.13‐1.41), *P* = 0.02] and IPTW weighted logistic regression [VE 0.83 (95% CI 0.03‐1.63), *P* = 0.04].

## 4 Discussion

Using multiple analytic approaches to adjust for bias associated with receipt of influenza vaccination in this observational study, we found that influenza vaccination was associated with lower prevalence of preterm birth (25‐34%) and low birthweight (24‐30%) in Cape Town during 2015‐2016. These associations are similar to those noted in some prior observational studies of birth outcomes following influenza vaccination and meta‐analyses of observational and experimental studies.^27^, ^37^, ^38^, ^39^ Delivery site, parity, number of antenatal visits, and smoking during pregnancy were also associated with preterm birth and low birthweight on multivariable analysis. Smoking during pregnancy is a well‐established source of reduced birthweight and preterm delivery.^40^, ^41^, ^42^, ^43^, ^44^ Delivery site was associated with birth outcomes in our study because two of the sites are referral centers that deliver high‐risk pregnancies. In the propensity score and IPTW analyses, number of antenatal visits was one of the indicators that was strongly predictive of vaccination and may be a source of residual bias in our analyses. Associations between vaccination and preterm birth or low birthweight were not significant among primigravidae in the same population. Possible reasons for this include insufficient sample size of primigravidae, unmeasured confounders in the overall population that may have biased our results, or a true lack of effect.

Although not significant in all models, influenza vaccination was associated with lower prevalence of stillbirth in our analyses in all pregnant women and primigravidae. A systematic review similarly found that vaccinated women had lower risk of stillbirth [RR 0.73 (95% CI 0.55‐0.96)] particularly in seasons where H1N1pdm09 predominated.^45^ The 2015 season in South Africa was H1N1pdm09 predominant but vaccine was delayed which may have limited our ability to measure an association between vaccination and prevention of stillbirth. Similarly, associations between vaccination and mean birthweight or SGA were not significant following adjustment for measured confounders, as seen in previous studies and reviews.^22^

We found that delays in influenza vaccine manufacturing in 2015 resulted in most vaccines being delivered after the peak of influenza virus transmission in the community. In 2016, influenza vaccines were delivered before the seasonal influenza virus peak. If the role of influenza vaccination in preventing adverse birth outcomes is due to prevention of influenza‐associated illness, we would expect that the protective effect of vaccination would have been more substantial or found only when vaccine was received before the peak of the influenza season in 2016 and not in 2015. However, campaign year was only significantly associated with lower prevalence of stillbirth in the final multivariable logistic regression models. Campaign year was not significantly associated with lower prevalence of preterm birth, low birthweight, or SGA

There are several limitations to this analysis including the retrospective design and the data sources. In South Africa, antenatal records and birth registers often lack sufficient data on pregnancies to adequately adjust for prior adverse birth outcomes. Difference in the timing and method of gestational age assessment, and the low proportion of women that initiate ANC during the first trimester, introduce uncertainty in ascertainment of gestational age which may impact assessment of preterm and SGA. Likewise, important predictors of infant weight including maternal education and pre‐pregnancy body mass index are not routinely collected or reported. Furthermore, the decision to receive influenza vaccine was not randomized. We identified a high proportion of women with non‐HIV chronic illness (24.2%) in this population; however, there was insufficient data to assess birth outcomes according to different disease types or severity of illness. The timing of influenza vaccination both in relation to the gestational age of the infant and the influenza transmission season is also important in understanding the potential impact of vaccination on birth outcomes. We adjusted for season of birth by determining if the infant was delivered before, during, or after the influenza season. This adjustment was balanced in the propensity score algorithm, but the standardized difference (0.323) following propensity score adjustment indicates that IPTW weighting may not have sufficiently adjusted for bias associated with the timing of vaccination.

In conclusion, in this retrospective observational study we identified statistically significant associations between antenatal influenza vaccination and reduced risk of low birthweight and preterm delivery. In future studies, it would be valuable to implement cluster randomization of vaccination, prospective data collection including maternal comorbidities and their severity, history of prior pregnancies, maternal BMI, education level, and socioeconomic status.

## Disclaimer

The findings and conclusions of this manuscript are those of the authors and do not reflect the official position of the US Centers for Disease Control and Prevention.

## Author Contribution

**Meredith McMorrow:** Conceptualization (equal); Formal analysis (lead); Methodology (equal); Resources (lead); Writing‐original draft (lead); Writing‐review & editing (lead). **Liza Rossi:** Data curation (equal); Project administration (lead); Supervision (lead); Writing‐original draft (supporting); Writing‐review & editing (supporting). **Susan Meiring:** Data curation (equal); Methodology (supporting); Project administration (equal); Supervision (equal); Writing‐original draft (supporting); Writing‐review & editing (supporting). **Katherine Bishop:** Data curation (equal); Project administration (lead); Supervision (supporting); Writing‐review & editing (supporting). **Raphaela Itzikowitz:** Data curation (supporting); Investigation (supporting); Project administration (supporting); Supervision (equal); Writing‐review & editing (supporting). **Washiefa Isaacs:** Data curation (supporting); Project administration (lead); Validation (supporting); Writing‐review & editing (supporting). **Faakhiera Stellenboom:** Data curation (lead); Validation (equal); Writing‐review & editing (supporting). **Sibongile Walaza:** Conceptualization (supporting); Methodology (supporting); Writing‐review & editing (supporting). **Orienka Hellferscee:** Investigation (supporting); Methodology (supporting); Supervision (equal); Writing‐review & editing (supporting). **Florette K Treurnicht:** Investigation (supporting); Supervision (supporting); Validation (equal); Writing‐review & editing (supporting). **Heather J. Zar:** Conceptualization (supporting); Data curation (supporting); Investigation (equal); Supervision (supporting); Writing‐review & editing (supporting). **Stefano Tempia:** Conceptualization (equal); Formal analysis (equal); Funding acquisition (equal); Methodology (equal); Writing‐review & editing (equal). **Cheryl Cohen:** Conceptualization (lead); Funding acquisition (equal); Methodology (lead); Supervision (equal); Writing‐original draft (equal); Writing‐review & editing (equal).

### Peer Review

The peer review history for this article is available at https://publons.com/publon/10.1111/irv.12836.
